# Management of Childhood Glaucoma Following Cataract Surgery

**DOI:** 10.3390/jcm11041041

**Published:** 2022-02-17

**Authors:** Anne-Sophie Simons, Ingele Casteels, John Grigg, Ingeborg Stalmans, Evelien Vandewalle, Sophie Lemmens

**Affiliations:** 1 Department of Ophthalmology, University Hospitals UZ Leuven, Herestraat 49, 3000 Leuven, Belgium; ingele.casteels@uzleuven.be (I.C.); ingeborg.stalmans@uzleuven.be (I.S.); evelien.vandewalle@uzleuven.be (E.V.); sophie.1.lemmens@uzleuven.be (S.L.); 2 Biomedical Sciences Group, Department of Neurosciences, Research Group Ophthalmology, KU Leuven, Herestraat 49, 3000 Leuven, Belgium; 3 Faculty of Medicine and Health, Save Sight Institute, The University of Sydney, 8 Macquarie St., Sydney, NSW 2000, Australia; john.grigg@sydney.edu.au

**Keywords:** childhood glaucoma, aphakia, pseudophakia, cataract surgery, lensectomy, management (or therapy), trabeculotomy, trabeculectomy, glaucoma drainage device, cyclodestruction

## Abstract

Glaucoma remains a frequent serious complication following cataract surgery in children. The optimal approach to management for ‘glaucoma following cataract surgery’ (GFCS), one of the paediatric glaucoma subtypes, is an ongoing debate. This review evaluates the various management options available and aims to propose a clinical management strategy for GFCS cases. A literature search was conducted in four large databases (Cochrane, PubMed, Embase, and Web of Science), from 1995 up to December 2021. Thirty-nine studies—presenting (1) eyes with GFCS; a disease entity as defined by the Childhood Glaucoma Research Network Classification, (2) data on treatment outcomes, and (3) follow-up data of at least 6 months—were included. Included papers report on GFCS treated with angle surgery, trabeculectomy, glaucoma drainage device implantation (GDD), and cyclodestructive procedures. Medical therapy is the first-line treatment in GFCS, possibly to bridge time to surgery. Multiple surgical procedures are often required to adequately control GFCS. Angle surgery (360 degree) may be considered before proceeding to GDD implantation, since this technique offers good results and is less invasive. Literature suggests that GDD implantation gives the best chance for long-term IOP control in childhood GFCS and some studies put this technique forward as a good choice for primary surgery. Cyclodestruction seems to be effective in some cases with uncontrolled IOP. Trabeculectomy should be avoided, especially in children under the age of one year and children that are left aphakic. The authors provide a flowchart to guide the management of individual GFCS cases.

## 1. Introduction

**Figure 1:**
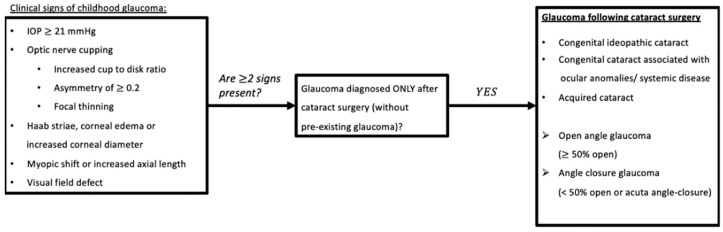
Glaucoma Following Cataract Surgery based on Childhood Glaucoma Research Network classification algorithm. Childhood: based on national criteria, <18 years old (USA); <16 years old (UK, Europe, UNICEF) (reproduced with permission from Grajewski, World Glaucoma Association Consensus Series 9: Childhood glaucoma, Kugler publications 2013 [5]). Abbreviations: IOP = Intra-Ocular Pressure.

Former terms used to describe childhood glaucoma, including ‘developmental’, ‘congenital’, or ‘infantile’, were often not clearly defined [1,2,3]. Therefore, the Childhood Glaucoma Research Network Classification recently developed a system for the classification of paediatric glaucoma to unify nomenclature (Figure 1) [3]. This review focuses on the subtype known as glaucoma following cataract surgery (GFCS), which accounts for 18% of childhood glaucoma [4].

After cataract extraction in early life, both aphakic and pseudophakic eyes are at high and lifelong risk for the development of glaucoma (GFCS, formerly known as aphakic or pseudophakic glaucoma). The incidence depends on duration of follow-up, age at time of surgery, corneal diameter, surgical techniques, and the definition of glaucoma among studies [6,7,8]. In the Infant Aphakia Treatment Study (IATS), a randomized clinical trial of 114 infants with unilateral congenital cataract who were aged 1 to 6 months at surgery, the incidence of GFCS was 22% after 10 years of follow-up [9]. Improved surgical techniques for childhood cataract extraction have reduced the incidence of angle-closure GFCS. The predominant type, accounting for 75 to 94% of GFCS, now has an open angle configuration [10]. Unlike angle-closure glaucoma, which is diagnosed in the early postoperative period, the incidence of open-angle glaucoma is known to rise with increasing postsurgical follow-up, and its presentation may occur any time after initially uneventful surgery [11]. Consequently, it is imperative that these patients receive lifelong follow-up in order to protect their vision.

The pathophysiological mechanism by which secondary glaucoma develops in these patients, who have a history of paediatric cataract, is still unclear and thought to be of multifactorial origin. A mechanical and a chemical theory have been hypothesized: (1) the mechanical support to the trabecular meshwork is lost after lensectomy and contributes to decreased trabecular spaces, potentially reducing outflow facility, or (2) the influence of chemical substances from the vitreous cavity and retained lens material may alter trabecular meshwork morphology and gene expression [12,13,14]. Further, early lensectomy and early use of high-dose steroids may also lead to structural alteration of the trabecular meshwork and associated impairment of normal angle maturation [11,15].

Age at time of lensectomy and microcornea are the two risk factors most commonly associated with a higher prevalence of GFCS [16]. The optimal timing of lensectomy is still under debate because the increased risk of developing GFCS must be balanced against the risk of irreversible deprivation amblyopia [10]. Until a few years ago, it was thought that pseudophakia was a protective factor for the development of GFCS [17]. However, selection bias in the possibility that children selected for intraocular lens placement have largely been those at lower risk for glaucoma may explain the observed lower incidence of glaucoma in pseudophakia in some initial small or non-randomised studies [16]. The IoLunder 2 Study and the Infant Aphakia Treatment Study (IATS) showed that the risk of glaucoma development at 5 and 10 years post lensectomy, respectively, is similar for aphakic and pseudophakic patients [9,18,19].

GFCS is often difficult to manage, and it is generally associated with a poor prognosis. The first-line treatment for most GFCS cases is medical [11,20]. When surgical intervention is indicated, the optimal surgical approach is subject to debate. Many studies reporting treatment outcomes of childhood glaucoma in general are available, but studies that focus on this particular glaucoma subtype are limited, resulting in a lack of consensus. This review summarizes the literature on the various medical and surgical options available for the management of GFCS, with the aim to suggest an appropriate management strategy for specific clinical GFCS cases. This paper provides a flowchart which may assist ophthalmologists treating GFCS patients in clinical decision-making.

## 2. Materials and Methods

This literature review was registered and approved by KU Leuven. In performing this review, the PRISMA statement (Preferred Reporting Items for Systematic Review and Meta-Analysis) was followed [21].

**Figure 2:**
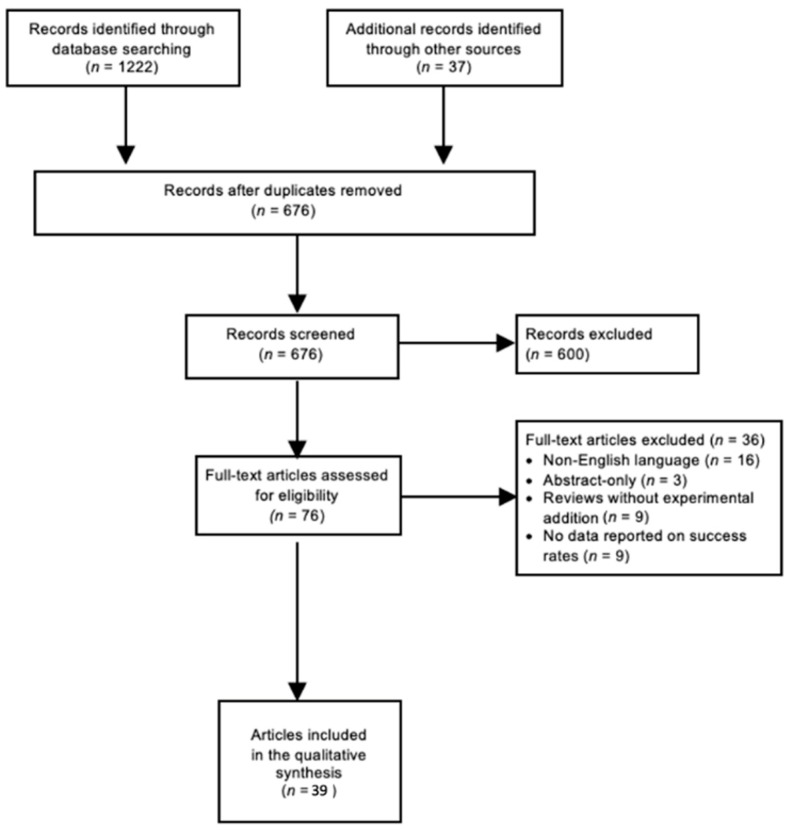
Literature search: PRISMA Consort flow diagram. According to THE PRISMA Statement 2009 [19]. Abbreviations: PRISMA = Preferred Reporting Items for Systematic Reviews and Meta-Analyses; *n* = amount of articles.

The databases Cochrane, PubMed, Embase, and Web of Science were systematically searched (from 1 January 1995 to 31 December 2021). A detailed search strategy for each database is presented in Appendix A. The individual references of each study were considered in order to identify additional relevant articles. Non-pertinent articles were rejected based on title and abstract screening. Thereafter, the full texts of the remaining articles were independently judged for eligibility by two independent reviewers (A.S., S.L.) according to the following inclusion criteria: the studies should (1) include eyes with GFCS; a disease entity as defined by the Childhood Glaucoma Research Network Classification (Figure 1), (2) report data on treatment outcomes, and (3) present follow-up data of at least six months after glaucoma therapy. Reviews that do not report original research results, non-English-language articles, and abstract-only articles were excluded. Inconsistencies were solved by consensus. The PRISMA flow diagram (Figure 2) gives details on the screening process. The authors performed a narrative synthesis because methodological heterogeneity precluded a meta-analysis. The Oxford Centre for Evidence-based Medicine classification was used to determine the Level Of Evidence (LOE) of the included papers [22].

## 3. Results

A total of 676 studies were screened using the described search strategy. At the end of the selection process, 39 articles were judged eligible for qualitative synthesis: 1 randomized controlled trial, 21 cohort studies, and 17 case series were included in the systematic review. In the following sections, summary tables of findings of included papers are provided. Medical therapy, angle surgery, trabeculectomy, glaucoma drainage device (GDD) implantation, and cyclodestructive procedures are mainly discussed. Five out of 39 studies described success rate with medication alone, 7 studies examined success rates of angle surgery, 8 studies examined success rates of trabeculectomy, 14 studies examined success rates of drainage implants, and 9 out of 39 studies examined success rates of cyclodestructive procedures.

### 3.1. Medical Treatment

Only five studies in which initial treatment was medical in all of the included eyes with GFCS clearly described their success rates with medication alone (Table 1) [23,24,25,26,27].

Success rates between the five available studies range from 17 to 73%, with those three reporting success rates of 40% and more having the largest study cohorts. Unlike PCG, which responds inadequately to medical therapy, these studies showed that long-term intra-ocular pressure (IOP) control can be reached with medication alone in some patients with GFCS. Medical therapy consists of topical medications and systemic medications, alone or in combination, in order to achieve the best possible result. Beta-blockers, carbonic anhydrase inhibitors, miotics and prostaglandin analogues are the classes commonly used for treatment of GFCS [23,24]. In a retrospective series of 32 eyes with GFCS, the addition of echothiophate iodide (EI) 0.125%, a miotic, in combination with other medications reduced IOP about 33% over long-term follow-up. The side-effects of EI were limited to transient redness that did not necessitate cessation of treatment [27].

### 3.2. Surgical Treatment

According to the literature, surgery is required in 27–83% of GFCS cases (Table 1) [23,24,25,26]. Repeat surgery and multiple modalities are often indicated to avoid or at least slow down further glaucoma progression. Similar ratios regarding the number of required repeat surgeries are reported, with 30–40% of eyes needing only one surgery and more than half of included study eyes needing two or more sequential surgical interventions [23,24]. One study documented the need of four or more procedures in 7% of eyes after a follow-up period of 18.7 years [24].

Included papers report on GFCS treated with angle surgery, trabeculectomy, GDD implantation, and cyclodestructive procedures.

#### 3.2.1. Angle Surgery

Data documenting this treatment modality in GFCS are limited and mostly presented by small retrospective cohorts with variably reported success rates (16–93%) (Table 2) [10,28,29,30,31,32,33]. Prior work found a success rate of only 16% after conventional angle surgery, including a 180-degee trabeculotomy or goniotomy [10]. This is in stark contrast with more recent studies in which success following angle surgery was achieved in the majority of eyes following a 360-degree trabeculotomy [28,29,30,32,33], with the most recent case series showing a success rate of 93% after a mean follow-up of 3.3 years [33]. The 360-degree trabeculotomy showed higher surgical success rates compared to conventional 180-degree goniotomy and trabeculotomy [29,32]. No visually devastating complications have been reported in the included studies.

Less favourable outcomes were reported in GFCS eyes with peripheral anterior synechiae [28,30].

#### 3.2.2. Trabeculectomy (+Antimetabolites)

Results of trabeculectomy + Mitomycin C in eyes with GFCS are variable but generally poor (Table 3) [10,23,34,35,36,37,38,39] with the largest cohort on this subject reporting a 25% success rate after a mean follow-up of 8.6 years [10].

Certain patient-related factors have shown to be significant risk factors for trabeculectomy failure, including aphakia and age younger than one year, especially when combined [35].

The use of antimetabolites can improve success rates but at the cost of increased risk of complications, including blebitis and endophthalmitis [37].

#### 3.2.3. Glaucoma Drainage Device Implantation

Most of the included studies report treatment outcomes of glaucoma drainage device implantation in eyes with GFCS with good success rates up to 95% (Table 4) [10,38,39,40,41,42,43,44,45,46,47,48,49,50,51,52]. Pakravan et al. demonstrated a 90% success rate after one year of follow-up following a glaucoma drainage device implantation as a primary procedure in eyes with GFCS. At five years, the success rate had fallen to 72% [49]. Other reports, analysing the effectiveness of GDDs in GFCS patients, noted similarly good success rates [40,41,48].

A prospective randomized clinical trial (RCT) found that GDD treatment outcome is superior to trabeculectomy. This RCT compared Ahmed glaucoma implant + MMC (AGI + MMC) with trabeculectomy + MMC (T + MMC) as the primary procedure for treatment of GFCS in children under 16 years of age. Each group consisted of 15 aphakic eyes, and although no statistically significant differences were found between both groups, the results were more favourable in the GDD + MMC group. The overall success rate was higher (87% vs. 73%), and the overall complication rate was lower (27% vs. 40%) in the AGI+MMC group versus T + MMC group, respectively [38]. Similarly, a retrospective study which revealed success rates of 44% after GDD implantation still shows more encouraging results when compared to a 24.6% success rate in patients who underwent trabeculectomy [10].

Similar to trabeculectomy, younger age at time of GDD surgery is associated with less favourable treatment outcomes. Nevertheless, in patients under two years of age, when compared to T + MMC, it was found that GDD implantation offered a significantly greater chance of successful IOP control. At the age of six, IOP was controlled in 19% in the trabeculectomy + MMC group versus in 53% in the GDD group [53]. A recent study demonstrated that the presence of persistent fetal vasculature (PFV) affects the outcome in a negative way; PFV-related cataracts showed a lower survival rate of the Ahmed glaucoma valve and a higher complication rate versus non-PFV-related cataracts [51]. Unlike for trabeculectomy, lens status is not consistently reported to be a significant risk factor for GDD failure. Patients with GFCS who have had multiple previous ocular surgeries may be at higher risk for tube failure, with better reported relative success rates in eyes with only one previous operation (83%) compared to those having had more than two previous operations (42%) [43].

Complications commonly described in the included studies after GDD implantation include suprachoroidal haemorrhage, choroidal detachment, hypotony, tube-corneal contact, and retinal detachment.

#### 3.2.4. Cyclodestructive Procedures

Moderate success rates have been reported after cyclodestructive procedures in GFCS eyes with uncontrolled (Table 5) [10,39,54,55,56,57,58,59,60]. The highest success rate (54%) was presented in a cohort of 35 aphakic or pseudophakic GFCS eyes, after a follow-up of 7.2 years [55].

In the study of Schlote et al., cyclodestruction showed better outcomes in older patients than in younger patients [60]. No effect of prior glaucoma interventions was found [53,55,56]. No significant differences in success rates between aphakic and pseudophakic eyes were found [55,57]. The only finding repeatedly associated with reduced outcomes was a higher pretreatment IOP; those eyes may need more sessions of cyclodestruction in order to control the IOP [55,57].

Postoperative hypotony, chronic uveitis, and rarely phthisis are complications reported after cyclodestruction [10,39,54,55,56,57,58,59,60]. Aphakic eyes with GFCS after endoscopic cyclophotocoagulation were at higher risk of postoperative complications, including retinal detachment, compared to eyes with other types of glaucoma [59].

## 4. Discussion

Medical therapy should be tried first in GFCS cases since long-term IOP control can be reached with medication alone in some cases. For example, Bhola et al. noted that 73% of patients achieved IOP with medication alone after a mean follow-up of 18.7 years [24]. The choice of medication varies between clinicians and depends on efficacy, potential adverse effects, cost, and availability across different health systems [5]. Kraus et al. found that EI had an impressive IOP-lowering effect in children with GFCS. Unfortunately, this medication was discontinued in 2021 and is currently no longer commercially available [27]. The decision to proceed to surgery should be a well-argued one, because younger age is frequently associated with reduced surgical outcomes; hence, medical therapy should be considered the initial strategy of choice in GFCS, possibly to bridge the time to surgery. On the other hand, topical IOP-lowering drugs have a higher potential for systemic adverse effects, and adherence to complex regimens is more difficult in young age groups [10]. When IOP control is inadequate, surgery should not be delayed because of fear of poor results.

Surgical treatment modalities for GFCS include angle surgery (trabeculotomy and goniotomy), GDD implantation, trabeculectomy with MMC, and cyclodestructive procedures. Given their normal life expectancy, children with GFCS may need multiple repeat interventions. Hence, the development of a long-term surgical strategy allowing a step-wise escalation of risk is strongly advisable. Selecting the most appropriate operation technique is crucial since the primary surgical intervention chosen for the child is often his or her best chance for long-term success.

**Figure 3:**
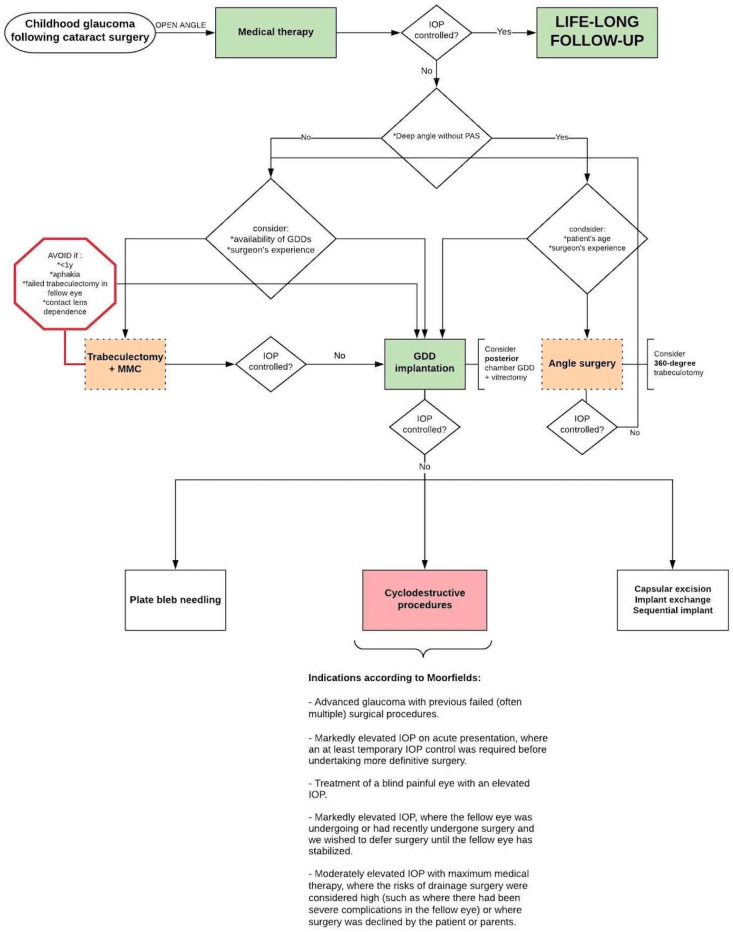
Suggested flowchart for the management of childhood GFCS with open-angle configuration (adapted with permission from Grigg, World Glaucoma Association Consensus Series 9: Childhood glaucoma, Kugler publications 2013 [16]). Abbreviations: IOP = Intra-Ocular Pressure; MMC = Mitomycin C; y = years; GDD = Glaucoma drainage device; PAS = peripheral anterior synechiae.

Some patient-related factors are associated with reduced outcomes of particular surgical procedures, making particular procedures more suitable than others for individual clinical cases. Since glaucoma surgery suffers from poor success rates in GFCS, knowledge about these patient-related factors affecting the outcomes helps in choosing the optimal approach for each individual patient. Considering the identified risk factors after reviewing the current literature, the authors suggest a flowchart for the management of GFCS (Figure 3). The flowchart was adapted from a previously published flowchart (Childhood Glaucoma, 9th Consensus Report of the World Glaucoma Association) [16].

It should be stressed that this is not intended as a pre-set algorithm that must be followed unconditionally as clinical decision-making will always be influenced by several factors (including surgeon preference/experience and local facilities/equipment availability). The next paragraphs may clarify the flowchart by summarising and interpreting the main findings of each surgical option.

Although angle surgery is more often reserved for cases of PCG, some recent studies have shown promising results in GFCS (Table 2); these studies are associated with the recent resurgence of interest in this treatment modality. This is not surprising since angle dysgenesis plays a role in the pathogenesis of GFCS and angle surgery addresses the physiological outflow pathways. In particular, 360-degree trabeculotomy maximises the therapeutic effect by providing both a temporal and nasal trabeculotomy at initial surgery, whether by two-site rigid probe or via microcatheter assisted suture placement. This technique is less invasive when compared to trabeculectomy, GDD implantation, and cyclodestructive procedures. Additionally, 360-degree trabeculotomy is beneficial because it spares the conjunctiva for potential future surgeries. Angle surgery was not mentioned in the previous flowchart of suggested management approach in 2013 (Childhood Glaucoma, 9th Consensus Report of the World Glaucoma Association) [16]. However, studies after 2013 showed good success rates in GFCS cases and the authors of this review suggest that a 360-degree trabeculotomy could be attempted as the primary surgical procedure in cases of relatively early-onset GFCS when the angle is deep and in the absence of peripheral anterior synechiae [20]. Some studies in the literature, mainly in the form of case reports, describe the performance of goniotomy with a 23 or 25 gauge straight cystotome or a Sinskey hook. These devices are much less expensive than other devices on the market for goniotomy such as Kahook blade, Goniotome, and Trabectome. It is a good option in resource-poor areas that cannot afford more expensive goniotomy devices [61,62,63,64,65,66].

Trabeculectomy has traditionally been the first choice of the remaining surgical options in childhood glaucoma, but it has shown limited success in GFCS (Table 3). Due to this limited success rates, the concern about bleb-related complications post trabeculectomy + MMC and due to the high success rates of up to 95% following GDD implantation in GFCS eyes (Table 4), there is a growing interest in selecting a GDD at primary surgery. Although large RCTs are lacking in this domain, the current literature does suggest that GDD implantation gives the best chance for long-term IOP control. Some studies put this technique forward as a good choice for primary surgery [38,44,48,49]. Where trabeculectomy was still considered as the primary procedure of choice in the previous flowchart (Childhood Glaucoma, 9th Consensus Report of the World Glaucoma Association) [16], the authors of this review suggest that glaucoma drainage implantation is preferred over trabeculectomy in GFCS cases. The complication of tube-cornea contact and corneal decompensation can be minimised by placing the tube in the sulcus in pseudophakic patients or pars plana with concomitant (or prior) vitrectomy in aphakic/pseudophakic patients [41].

Cyclodestructive procedures (Table 5), plate bleb needling, and exchange or sequential implant have proven to be effective in patients with uncontrolled IOP after a GDD implantation [67,68,69]. Cyclodestruction is generally considered when other options have failed. Although initially reserved for end-stage glaucomatous eyes in which multiple procedures have failed, indications for this procedure have expanded, and it can be considered an initial surgical approach in selected cases (Figure 3, indications according to Moorfields).

The aetiology of GFCS is largely not understood and thought to be multifactorial in origin. A significant reduction in Schlemm’s canal (SC) size and loss of SC dilation during physiologic accommodation in children with GFCS has recently been demonstrated, suggesting that targeting SC may potentially offer a new management approach [70] 3. Future research directed at better understanding the underlying aetiology is necessary since such an understanding may have implications for the clinical management of GFCS.

One of the major strengths of this review is that it specifically focuses on the management of the glaucoma subtype GFCS. Many reports in the literature offer a comparison of different procedures for childhood glaucoma in general; however, the mix of diagnoses of subjects differs between studies and different aetiologies of glaucoma do not respond in the same manner to a particular surgical intervention. For that reason, the authors chose to extract and analyse the outcomes separately for patients with GFCS. However, it must be noted that the differing study results are limited by their retrospective nature, varying study populations (including patient age and the severity of glaucoma), varying techniques and devices, and varying number of previous surgeries as well differences in the definitions of success and failure.

## 5. Conclusions

Although medical therapy is usually the first-line treatment for GFCS, multiple surgical procedures are often required to adequately control the condition. It might be worth trying a 360-degree trabeculotomy before proceeding to glaucoma drainage device implantation, since this technique offers good results and is less invasive. Glaucoma drainage device implantation seems to give the best chance for long-term IOP control in childhood GFCS and some studies put this technique forward as a good choice for primary surgery. Cyclodestruction seems to be effective in some GFCS cases with uncontrolled IOP after a glaucoma drainage device implantation. Trabeculectomy offers poor success rates in children with GFCS, especially in children under the age of one year and children that are left aphakic. The authors provide a flowchart to guide the management of individual GFCS cases.

## Figures and Tables

**Table 1:** Relevant studies involving success rates with medications alone and the need for surgical treatment in GFCS eyes.

Author, Year, Study Design (LOE) Reference	Inclusion and Exclusion Criteria	Mean Pre-Treatment IOP ± SD (mmHg)	Mean Age at GDx ± SD (Years)	*n* Eyes (a-p)	Success Criteria	Success Rate with Medications Alone (%) *Number of Drugs* (*% of Cases*)	Need for Surgery (%) *Number of Operations* (*% of Cases*)	Mean (*) Follow-Up ± SD (Years)
Bhola et al. (2006), retrospective cohort study (2b) [24]	Inclusion: IOP > 25 mmHg following congenital cataract surgery. Exclusion: Lensectomy > 10 y; Ocular conditions; Systemic syndromes; Traumatic cataracts; PCG; Follow-up < 1 y.	32 ± 6	7	55 (55-0)	IOP ≤ 25 mmHg	73 *1*–*2* (*36*) *3* (*33*) ≥*4* (*31*) Β-blockers, Cholinergic agents, Adrenergic agonists, Carbonic anhydrase inhibitors, Prostaglandin analogues.	27 *1* (*40*) *2*–*3* (*53*) *4*–*6* (*7*) Goniotomy, trabeculotomy, trabeculectomy ± MMC, GDD implantation, cyclodestruction.	18.7 ± 8
Comer et al. (2011), retrospective cohort study (2b) [25]	Inclusion: Persistently elevated IOP, and/or evidence of corneal enlargement, and/or optic disc cupping. Exclusion: Primary intraocular lens implantation; Lensectomy > 1 y; Pre-existing glaucoma; Ocular conditions	28.6 ± 5.9	2.6	18 (18-0)	IOP ≤ 20 mmHg	17 *Not further specified*	83 ≥ *2* (*61*) GDD implantation, trabeculectomy + MMC, goniotomy, cyclodestruction.	6.5
Kraus et al. (2015), retrospective case series (4) [27]	Inclusion: Aphakic and pseudophakic children < 18 y from 1992 to 2013 EI Exposure Glaucoma following cataract surgery was defined according to the consensus established by The Childhood Glaucoma Research Network Classification Exclusion: -	32.1	3.2	32 (27-5)	IOP-lowering effect of: 5–10 mmHg <5 mmHg	41 37 EI	12.5 rabeculotomy, GDD implantation	7.88
Baris et al. (2019), retrospective cohort study (2b) [23]	Inclusion: IOP > 25 mmHg following congenital cataract surgery. Exclusion: Pre-existing glaucoma; Anterior segment dysgenesis; Severe microphthalmia; Previous laser.	29.8 ± 14.8	1 ± 2.1	40 (40-0)	IOP < 21 mmHg	50 *1* (*75*) *2* (*20*) *3* (*5*) First choice: dorzolamide-timolol combination; Second choice: prostaglandin analogues.	50 *1* (*30*) *2* (*20*) ≥*3* (*50*) First choice: trabeculectomy +MMC (0.2 mg/mL for 4 min); Second choice: GDD implantation; Third choice: cyclodestruction	6.6 ± 2.6
Spiess et al (2020), retrospective cohort study (2b) [26]	Inclusion: Paediatric patients with GFCS from 1996 to 2016; Glaucoma following cataract surgery was defined according to the consensus established by The Childhood Glaucoma Research Network Classification Glaucoma Association in 2018) as intraocular pressure (IOP) greater than 21 mmHg with associated anatomical optic disc changes or other signs of progressive myopia. Exclusion: Acquired cataracts, trauma, anridia, Lowe syndrome, an age of 2 years and older with congenital cataract surgery, and previous/concomitant ocular hypertension or primary intraocular lens (IOL) implantation	29.1 ± 5.6	-	58 (47-9)	IOP < 21 mmHg with or without medication	41 40% monotherapy 60% combination therapy The most frequently prescribed drugs were beta-blockers (82%), followed by carbonic anhydrase inhibitors, prostaglandins, and alpha-2 adrenergic agonists.	59 70% tube implantation 24% trabeculectomy 6% peripheral iridotomy	4.6 *

Abbreviations: GDx = Glaucoma diagnosis; LOE = level of evidence; SD = Standard Deviation; GFCS = Glaucoma Following Cataract Surgery; a = aphakic; p = pseudophakic; mg = milligram; mL = millilitre; min = minutes; GDD = Glaucoma Drainage Device; IOP = Intra-Ocular Pressure; PCG = Primary Congenital Glaucoma; MMC = Mitomycin C; IOL = Intra-Ocular Lens, *n* = amount; (*) = or median: EI = Echothiophate iodide.

**Table 2:** Relevant studies involving angle surgery in GFCS eyes.

Author, Year, Study Design (LOE) Reference	Inclusion and Exclusion Criteria	Mean Pre-Treatment IOP ± SD (mmHg)	Mean Age at GDx ± SD (y)	Mean (* Median) Age at Glaucoma Surgery ± SD (y)	*n* Eyes (a-p)	Procedure	Success Criteria	Success Rate (%)	Mean (*) Follow-Up ± SD (y)	Factors Affecting Treatment Outcomes *% of Eyes That Had Prior Glaucoma Surgery*
Chen et al. (2004), retrospective cohort study (2b) [10]	Inclusion: IOP > 25 mmHg following congenital cataract surgery; Lensectomy < 20 y. Exclusion: Pre-existing glaucoma; History of trauma; Intraocular neoplasm; Radiation therapy; Anterior uveitis; anterior segment dysgenesis; Ocular syndromes; PCG; Corticosteroid use before lensectomy.	-	-	-	24 (24-0)	Goniotomy and rigid-probe Trabeculectomy Ab externo	IOP ≤ 21 mmHg with and without medications and with no need for further surgery.	16	8.6 ± 7.6	- *Not specified*
Bothun et al. (2010), retrospective cohort study (2b) [28]	Inclusion: Medically refractory glaucoma; IOP > 25 mmHg following congenital cataract surgery; Changes in corneal diameter or clarity; Increased axial length; Increased optic-nerve cupping; A combination of the above. Exclusion: Anterior segment dysgenesis; Microcornea (corneal diameter < 9.5 mm) Pre-existing glaucoma; Follow-up < 1 y.	35 ± 10	-	3.1	14 (14-0)	Goniotomy and/or rigid-probe trabeculotomy (lateral 180° initially, repeat nasal 180°) Ab externo	IOP ≤ 24 mmHg with or without topical medication; a lack of sight-threatening complication: and avoidance of trabeculectomy or GDD.	57 (after a mean of 1.4 angle surgeries per eye) 43 (after a single procedure)	4.7	Eyes with initial trabeculotomy required fewer procedures than those with an initial goniotomy. *0%*
Beck et al. (2011), retrospective case series (4) [29]	Inclusion: Glaucoma following congenital cataract surgery, diagnosed < 3 y. Exclusion: Three or more clock hours of peripheral anterior synechiae; Iridocorneal adhesions; Pre-existing glaucoma.	33.0 ± 7.2	-	* 5.0	4 (4-0)	360-degree suture trabeculotomy Ab externo	IOP < 22 mmHg with and without medication.	75	* 1.6	- *0%*
Dao et al. (2014), retrospective case series (4) [30]	Inclusion: Medically refractory glaucoma following lensectomy Exclusion: Pre-existing glaucoma; Anterior segment anomalies; Extensive synechiae; PCG; Surgical interventions other than planned	35.4 ± 4.7	-	3.1	13 (10-3) All open angle	360° microcatheter trabeculotomy Ab externo	IOP ≤ 22 mmHg with 30% reduction, without disease progression, oral glaucoma medications or additional glaucoma surgery.	62	1.4	- 0%
Lim et al. (2017), retrospective case series (2b) [32]	Inclusion: Medically refractory glaucoma following cataract surgery; Micro-assisted trabeculotomy as initial surgical procedure. Exclusion: Prior glaucoma surgery (laser or incisional); Suture trabeculotomy; Second eyes receiving subsequent 360-degree trabeculotomy; Coexisting ocular or systemic syndrome.	31.5 ± 7.5	3.3 ± 3.9	5.6 ± 5.6	25 (19-6)	360° microcatheter trabeculotomy Ab externo	IOP ≤ 22 mmHg and 20% reduction without additional glaucoma surgery or devastating complication.	72	2.7 ± 2.2	Lens status (*p* = 0.88) *0%*
El Sayed et al. (2020), prospective cohort study (2b) [31]	Inclusion: Children ≤ 14 years who required surgery for GFCS. Exclusion: Synechial angle closure over ≥90°; Requirement of combined procedures; Previous procedures other than lensectomy or IOL implantation; Eyes in which the trabeculotomy involved < 180° of Schlemm’s canal	26.8 ± 8.2	-	5.73 ± 1.79	29 (16-13)	Two-site rigid probe trabeculotomy 180–360° Ab externo	IOP < 23 mmHg or 30% IOP reduction, on the same or fewer number of medications at 1 year, without the need for another glaucoma procedure	89.6 (51.7% without medications)	1.4	No significant difference in the final IOP of aphakic and pseudophakic eyes. *0%*
Rojas et al. (2020), retrospective case series [33]	Inclusion: Children ≤ 18 years who underwent trabeculotomy January 2013 and July 2019 Exclusion:-	27.1 ± 7	-	7.8 ± 5.8	15 (12-3)	360° microcatheter trabeculotomy Ab externo	5 < IOP < 20 without additional surgery	93	3.3 ± 2.4	- *0%*

Abbreviations: GDx = Glaucoma diagnosis; LOE = level of evidence; SD = Standard Deviation; GFCS = Glaucoma Following Cataract Surgery; a = aphakic; p = pseudophakic; min = minute; GDD = Glaucoma Drainage Device; IOP = Intra-Ocular Pressure; PCG = Primary Congenital Glaucoma; mm = millimetres; y = years; (*) = or median; ° = degree.

**Table 3:** Relevant studies involving trabeculectomy in GFCS eyes.

Author, Year, Study Design (LOE) Reference	Inclusion and Exclusion Criteria	Mean Pre-Treatment IOP ± SD (mmHg)	Mean Age at GDx ± SD (y)	Mean (*) Age at Glaucoma Surgery ± SD (y)	*n* eyes (a-p)	Antimetabolites	Success Criteria	Success Rate (%)	Mean (*) Follow-Up ± sd (y)	Factors Affecting Treatment Outcomes *% of Eyes That Had Prior Glaucoma Surgery*
Beck et al. (1998), retrospective case series (4) [35]	Inclusion: Glaucoma (not further defined) following congenital cataract surgery; ≤17 y. Exclusion: -	35.8 ± 8.0	-	7.6	9 (7-2)	MMC 0.25 mg/mL for 5 min	IOP ≤ 22 mmHg with and without medication, no evidence of glaucoma progression, no further need of glaucoma surgery.	78	2.5 ± 1.3	Age < 1 y (*p* = 0.0005) Aphakia (*p* = 0.0364) Anterior segment dysgenesis/aniridia (*p* = 0.49) *Not specified*
Wallace et al. (1998) retrospective cohort study (2b) [39]	Inclusion: Glaucoma (not further defined) after congenital cataract surgery; Need of glaucoma surgery < 18 y. Exclusion: PCG.	35.9	6.1	8.7	13 (13-0)	MMC 0.2 to 0.4 mg/mL for 4 min	IOP ≤ 25 mmHg without medications and IOP ≤ 21 mmHg with medications.	62	4.2	- *Not specified*
Azuara-Blanco et al. (1999), retrospective case series (4) [34]	Inclusion: Glaucoma (not further defined) following congenital cataract surgery; Aphakia; <18 y. Exclusion: -	35.7 ± 10.5	-	5.7 ± 5.0	8 (8-0)	MMC 0.4 mg/mL for 1–5 min	Absolute success: IOP < 21 mmHg with no antiglaucoma medications, with apparently stable glaucoma and absence of severe complications. Relative success: No performance of or recommendation for further glaucoma surgery and absence of severe complications.	0 33	1.6 ± 1.2	Phakic cases (PCG) seemed to have a better outcome than aphakic cases. 12.5%
Freedman et al. (1999), retrospective case series (4) [36]	Inclusion: Glaucoma refractory to maximum medical treatment, prior angle or filtration surgery (including goniotomy, trabeculotomy, or trabeculectomy) or both; <17 y; Aphakia. (*Note: 2 aphakic glaucomatous eyes were uveitic, one aniridic and one case had PHPV*) Exclusion: -	35.6	-	7.2	7 (7-0)	MMC 0.4 mg/mL for 3–5 min and postoperative 5-fluorouracil, laser suture or both	4 mmHg < IOP < 16 mmHG without further glaucoma surgery or devastating complication.	29	1.9	Age < 1 y and aphakia (vs. phakic status in PCG and JOAG), taken together. (*p* = 0.013) The addition of postoperative 5-fluorouracil and suture lysis did not provide improvement and may have increased complication rate. *42.8%*
Mandal et al. (2003), retrospective case series (4) [37]	Inclusion: Glaucoma with aphakia or pseudophakia after congenital cataract surgery. Exclusion: -	34.2 ± 8.9	9.6	9.9 ± 9.0	23 (21-2)	MMC 0.4 mg/mL for 3 min	Complete success: 6 mmHg < IOP < 21 mmHg without medication. Qualified success: 6 mmHg < IOP < 21 mmHg, with or without 1 topical medication, no further need of glaucoma surgery, and no visually devastating complication.	37 58	2.0 ± 1.5	- *Not specified*
Chen et al. (2004), retrospective cohort study (2b) [10]	Inclusion: IOP > 25 mmHg following congenital cataract surgery; Lensectomy < 20 y. Exclusion: Pre-existing glaucoma; History of trauma; Intraocular neoplasm, radiation therapy, anterior uveitis, anterior segment dysgenesis, ocular syndromes; PCG: Corticosteroid use before lensectomy.	-	-	-	61 (61-0)	MMC (*n* = 43) 5-fluorouracil (*n* = 17) None (*n* = 1)	IOP ≤ 21 mmHg with and without medications and no need for further surgery.	25	8.6 ± 7.6	- *Not specified*
Pakravan et al. (2007), prospective randomized clinical trial (1) [37]	Inclusion: Medically unresponsive glaucoma (not further defined) following congenital cataract surgery, <16 y. Exclusion: Any history of ocular surgery other than anterior lensectomy/vitrectomy; Congenital cataract in the setting of PFV or intrauterine infections; Follow-up < 6 m.	31 ± 10.7	-	9.1 ± 4.1	15 (15-0)	MMC 0.02% for 2 min	Absolute success: 5 mmHg ≤ IOP < 21 without medications. Qualified success: 5 mmHg ≤ IOP < 21 with ≤2 medications. Overall success: absolute + qualified success. mmHg ≤ IOP < 21	33 40 73	1.2 ± 0.9	- *0%*
Baris et al. (2019), retrospective cohort study (2b) [23]	Inclusion: IOP > 25 mmHg following congenital cataract surgery. Exclusion: Pre-existing glaucoma; Independent risk factors for glaucoma development, such as anterior segment dysgenesis or severe microphthalmia; Previous laser.	29.8 ± 14.8	1 ± 2.1	-	20	MMC 0.2 mg/mL for 4 min	Complete success: IOP < 21 mmHg without medication. Qualified success: IOP < 21 mmHg with and without medication.	5 30	6.6 ± 2.6	- *0%*

Abbreviations: GDx = glaucoma diagnosis; LOE = level of evidence; SD = Standard Deviation, GFCS: Glaucoma Following Cataract Surger; a = aphakic; p = pseudophakic; (*): median; mg = milligram; mL = millilitre; min = minutes; GDD = Glaucoma Drainage Device; IOP = Intra-Ocular Pressure; PCG = Primary Congenital Glaucoma; JOAG = Juvenile open-angle glaucoma; MMC = Mitomycin C; *n* = amount; PHPV = persistent hyperplastic primary vitreous; y = years.

**Table 4:** Relevant studies involving glaucoma drainage device implantation in GFCS eyes.

Author, Year, Study Design (LOE) Reference	Inclusion and Exclusion Criteria	Mean Pre-Treatment IOP ± SD (mmHg)	Mean Age at GDx ± SD (y)	Mean (*) Age at Glaucoma Surgery ± SD (y)	Number of Eyes (a-p)	Device ± Antimetabolites	Success Criteria	Success Rate (%)	Mean (*) Follow-Up ± SD (y)	Factors Affecting Treatment Outcomes *% of Eyes That Had Prior Glaucoma Surgery*
Donahue et al. (1997), retrospective cohort study (2b) [43]	Inclusion: Glaucoma (not further defined) after lensectomy Indications for lensectomy were congenital cataract (*n* = 7), PHPV (*n* = 2) and Lowe syndrome (*n* = 1); Exclusion: -	33	-	-	10 (9-1)	Baerveldt 350 mm	Complete success: No further reoperation, no decrease in vision, and IOP at last follow-up < 21 mmHg, without complication not associated with tube failure. Qualified success: With and without medication necessary to bring IOP < 21 mmHg, with or without complication not associated with tube failure.	40 70	1.6	It appeared that the aphakic patients who has had multiple previous procedures were at higher risk for shunt failure. *40%*
Wallace et al. (1998), retrospective cohort study (2b) [39]	Inclusion: Glaucoma (not further defined) after congenital cataract surgery; Need of glaucoma surgery < 18 y. Exclusion: PCG.	35.9	6.1	8.7	9	Molteno	IOP ≤ 25 mmHg without medication and IOP ≤ 21 mmHg with medication.	67 at 6 m 33 at 1 y	4.2	- *Not specified*
Englert et al. (1999), retrospective case series (4) [45]	Inclusion: <18 y; Medically uncontrolled glaucoma or uncontrolled despite previous glaucoma surgery (goniotomy, trabeculotomy, trabeculectomy ± antimetabolites, and/or cycloablative procedures); Exclusion: -	32.8 ± 7.5	-	-	7 (7-0)	Ahmed S-2 model in the superotemporal quadrant	IOP ≤ 21 mmHg without medication without further surgery without visually devastating complication	86	1.0 ± 0.7	Previous cycloablation was not a significant risk factor for failure. *14.2%*
Chen et al. (2004), retrospective cohort study (2b) [10]	Inclusion: IOP > 25 mmHg following congenital cataract surgery; Exclusion: Pre-existing glaucoma; History of trauma; Intraocular neoplasm, radiation therapy, anterior uveitis, anterior segment dysgenesis, ocular syndromes; PCG.	-	-	-	34	Ahmed (32 eyes) Molteno (2 eyes)	IOP ≤ 21 mmHg with and without medications and no need for further surgery.	44	8.6	- *Not specified*
Chen et al. (2005), retrospective case series (4) [42]	Inclusion: Aphakic glaucoma (not further defined) after congenital cataract surgery; <18 y. Exclusion: -	38.1 ± 6.4	-	4.9 ± 6.5	19	Ahmed S-2 model	IOP ≤ 22 mmHg with or without medications, without further surgery, without visually devastating complications	68 (75 if GDD implantation was the initial surgery)	2.2 ± 1.8	- *57.9%*
Kirwan et al. (2005), retrospective case series (4) [46]	Inclusion: Paediatric aphakic glaucoma (diagnosis of glaucoma was mainly based on changes in optic disc and IOP, not further defined); Uncontrolled glaucoma by medical therapy or other forms of surgery (cycloablation and/or trabeculectomy). Exclusion: -	31.1	-	8	19	Ahmed S-2 model In 10 eyes: +MMC 0.5 mg/mL for 3 min	IOP ≤ 15 with and without medical therapy.	95	2.7	- *47.4%*
Pakravan et al. (2007), prospective randomized control trial (1) [38]	Inclusion: Medically unresponsive glaucoma (not further defined) following congenital cataract surgery; <16 y. Exclusion: Any history of ocular surgery other than anterior lensectomy/vitrectomy; Congenital cataract in the setting of PFV or intrauterine infections; Follow-up < 6 m.	31 ± 7.5	-	10.9 ± 5.1	15 (15-0)	Ahmed + MMC 0.2 mg/mL for 2 min	Absolute success: 5 mmHg ≤ IOP < 21 mmHg without medications. Qualified success: 5 mmHg ≤ IOP < 21 mmHg with ≤ 2 medications. Overall success: absolute + qualified success.	20 67 87	1.1 ± 0.8	- *0%*
O’Malley Schotthoefer et al. (2008), retrospective cohort study (2b) [48]	Inclusion: Medically refractory glaucoma (not further defined) after cataract surgery. Exclusion: -	36	-	4.3 *	41 (38-3)	Ahmed S-2 or FP-7 (*n* = 16) Baerveldt (*n* = 22) Molteno (*n* = 3) All in superotemporal quadrant. PP + V in 5 eyes	IOP ≤ 21 mmHg without medication, without further surgery, without visually devastating complications.	90 at 1 y 82 at 2 y 55 at 10 y	0.5 *	Better reported outcomes with Ahmed valve implantation in aphakic glaucoma than refractory PCG, no statistically significant difference in Kaplan Meier. *0%*
Banitt et al. (2009), retrospective cohort study (2b) [41]	Inclusion: <18 y; Uncontrolled glaucoma associated with aphakia or pseudophakia; PCG (*n* = 1); Secondary glaucoma (*n* = 29), Glaucoma following cataract surgery *(n* = 21), Glaucoma post ocular trauma (*n* = 5) Glaucoma associated with aniridia (*n* = 3). Exclusion: Prior aqueous shunt surgery with anterior tube insertion.	32.9 ± 7.9	-	6.9 ± 5.0	30 (24-6)	Baerveldt PP + V in all eyes	5 mmHg ≤ IOP < 21 mmHg, with and without medications, and without visually devastating complication or further surgery.	85 at 1 y 81 at 2 y 72 at 3 y	2.5 ± 2.2	Lens status (aphakia vs. pseudophakia) had comparable IOP results (*p* = 0.77). *Not specified*
Balekudaru et al. (2014), retrospective cohort study (2b) [40]	Inclusion: Medically uncontrolled glaucoma (not further defined) in aphakia and pseudophakia; <18 y; Results of only the first implant were included in eyes that underwent surgery with more than one implant. Exclusion: -	35.86 ± 9.57	-	-	47	Ahmed S-2 or FP-7 model in the superior-temporal quadrant	Complete success: 6 mmHg ≤ IOP ≤ 18 mmHg with and without medication. Qualified success: 6 mmHg ≤ IOP ≤ 18 mmHg, without visually devastating complication.	95 at 1 y 86 at 2 y	-	No significant differences in outcomes between the two Ahmed valve models. *76,6%*
Elshatory et al. (2016), retrospective case series (4) [44]	Inclusion: Aphakic glaucoma (not further defined) The causes of aphakia were congenital cataract extraction (*n* = 12), post-traumatic cataract extraction (n = 1) and Peter’s anomaly (*n* = 1); GDD implantation was the initial procedure; Exclusion: Follow-up < 6 m.	33.9 ± 10.9	-	9.2 ± 5.7	14	Ahmed (36%) Baerveldt (64%) PP+V in all eyes	Improved postoperative IOP control without any intra- or postoperative complications.	Average decrease in IOP of 51%	1.0	- *0%*
Pakravan et al. (2019), retrospective case series (4) [49]	Inclusion: Aphakic glaucoma (not further defined) following cataract extraction; Ahmed glaucoma valve implantation as primary procedure. Exclusion: Follow-up < 6 m; Prior cyclodestructive procedures.	28.9 ± 6.1	-	9.9 ± 5.6	33	Ahmed FP-7	5 mmHg < IOP < 21 mmHg with or without medication.	90 at 1 y 72 at 5 y	4.1 ± 3.4	Better reported outcomes with Ahmed valve implantation in aphakic glaucoma than refractory PCG. *0%*
Geyer et al. (2021) Retrospective case series (4) [52]	Inclusion: Paediatric patients with GFCS: congenital cataract Ahmed glaucoma valve implantation between 2007 and 2018 Exclusion: -	35.8 ± 7.4	-	6.6 *	41	Ahmed	IOP ≤ 22 mmHg without glaucoma reoperations and without significant complications	95 at 1 y 90 at 2 y 83 at 5 y 73 at 7 y	5 *	- *17%*
Spiess et al. (2021), retrospective cohort study (2b) [51]	Inclusion: Paediatric patients with GFCS from 1996 to 2016; Glaucoma following cataract surgery was defined according to the consensus established by The Childhood Glaucoma Research Network (World Glaucoma Association in 2018) as intraocular pressure (IOP) greater than 21 mmHg with associated anatomical optic disc changes or other signs of progressive myopia. Exclusion: Acquired cataracts, trauma, anridia, Lowe syndrome, an age of 2 years and older with congenital cataract surgery, and previous/concomitant ocular hypertension or primary intraocular lens (IOL) implantation.	32.66 ± 6.73	Median 2.9y after cataract surgery	2	29 (23 aphakic, 6 pseudophakic) 41% PHPV 59% non-PHPV	Ahmed: model FP7, model S2, and model FP8	IOP < 21 mmHg with or without medication. PHPV Non-PHPV	37.5 at 1 y, 28.1 at 5 y 88.2 at 1 y, 71.9 at 5 y	7.5	Eyes with PHPV and GFCS followed by AGV implantation had a higher number of complications and a decreased probability of success compared to the nonpersistent foetal vasculature group. Both groups achieved a significant decrease in intraocular pressure. *Not specified*

Abbreviations: GDx = glaucoma diagnosis; LOE = level of evidence; SD = Standard Deviation, GFCS: Glaucoma Following Cataract Surger; a = aphakic; p = pseudophakic; (*): median; mg = milligram; mL = millilitre; min = minutes; GDD = Glaucoma Drainage Device; IOP = Intra-Ocular Pressure; PCG = Primary Congenital Glaucoma; JOAG = Juvenile open-angle glaucoma; MMC = Mitomycin C; *n* = amount; PHPV = persistent hyperplastic primary vitreous; y = years; C/D = cup to disk ratio; PP+V: Posterior placement + vitrectomy.

**Table 5:** Relevant studies involving cyclodestruction in GFCS eyes.

Author, Year, Study Design (LOE) Reference	Inclusion and Exclusion Criteria	Mean Pre-Treatment IOP ± SD (mmHg)	Mean Age at GFCS Diagnosis ± SD (y)	Mean (* Median) Age at Glaucoma Surgery ± SD (y)	Number of Eyes (a-p)	Procedure	Success Criteria	Success Rate (%)	Mean (* Median) Follow-Up ± SD (y)	Factors Affecting Treatment Outcomes *% of Eyes That Had Prior Glaucoma Surgery*
Wallace et al. (1998), retrospective cohort study (2b) [39]	Inclusion: Glaucoma (not further defined) following cataract surgery; Need of surgery < 18 y. Exclusion: PCG.	35.9	6.1	8.7	4	ECP	IOP ≤ 25 mmHg without medications and IOP ≤ 21 mmHg with medications.	50	4.2	- *Not specified*
Neely and Plager (2001), retrospective cohort study (2b) [59]	Inclusion: 51 ECP procedures performed on 36 eyes of 29 paediatric patients with glaucoma. Aphakic glaucoma (*n* = 19, 53%) PCG (*n* = 10), Sturge-Weber syndrome (*n* =2 ), Anterior segment dysgenesis (*n* = 1) Microphthalmia (*n* = 1) Note: In addition to the 19 eyes with aphakic glaucoma after removal of congenital cataracts, 3 additional ones	35.06 ± 8.55	-	4.90 ± 4.17	22 aphakic eyes (19 GFCS, 3 PCG)	ECP	IOP ≤ 21 mmHg, with and without antiglaucoma medications.	50	1.6 ± 1.6	Aphakic patients may have an increased risk of significant postoperative complications, such as retinal detachment. *Not specified*
Kirwan et al. (2002), retrospective cohort study (2b) [58]	Inclusion: <18 y; Aphakic glaucoma (*n* = 34, 45%) and PCG (*n* = 26, 35%), other types of glaucoma (aniridia, anterior segment dysgenesis, uveitic glaucoma, Sturge-Weber, silicone-oil-associated glaucoma, naevus- or Ota-associated glaucoma, secondary angle-closure glaucoma) Note: Of 28 eyes that underwent primary cyclodiode, 23 were aphakic; Advanced glaucoma with previous failed surgical procedures; Markedly elevated IOP on acute presentation; Blind, painful eyes; Markedly elevated IOP, where the fellow eye had recently undergone surgery; Moderately elevated IOP with maximum therapy, where risks of drainage surgery were considered high or where surgery was declined by the patient or parents. Exclusion: Follow-up < 1 y.	32.0 ± 6.4	-	7.4	34	TDLC (300°)	IOP < 22 mmHg or reduction by 30%, with and without antiglaucoma medications.	42 at 1 y	1.8	Aphakic eyes had a more sustained IOP control than phakic eyes (PCG, aniridia, anterior segment dysgenesis, uveitic glaucoma, Sturge-Weber, silicone-oil-associated glaucoma, naevus- or Ota-associated glaucoma, secondary angle-closure glaucoma). Aphakic patients had a 42% IOP control at one year versus 14% in phakic eyes. (*p* < 0.001 log rank test). Success rate is lower than in adults, and younger eyes may recover from treatment more rapidly. *Not specified*
Autrata and Lokaj (2003), retrospective cohort study (2b) [54]	Inclusion: Glaucomatous eyes that underwent TDLC aphakic glaucoma (*n* = 26), PCG (*n* = 21), Other glaucomas (uveitic glaucoma, secondary angle closure, Sturge-Weber, aniridia); Advanced glaucoma with previous failed surgical procedures; Markedly elevated IOP where an IOP control was required before undertaking definitive surgery; Moderately elevated IOP with maximum medical therapy where the risks of drainage surgery were considered high; Blind, painful eyes with an elevated IOP. Exclusion: Follow-up < 1 y.	34.08 ± 7.13	-	6.1	26	TDLC (300°)	IOP ≤ 21 mmHg, with and without adjunctive antiglaucoma medications.	47 at 1 y	5.6 ± 2.8	Aphakic patients had a more sustained IOP-lowering response after their first treatment session. Of aphakic eyes, 47% had IOP control at one year versus 19% of the phakic eyes (PCG, uveitic glaucoma, secondary angle closure, Sturge-Weber, aniridia). The data suggest that multiple repeated cyclodiode treatments may still have an IOP-lowering effect. *Not specified.*
Chen et al. (2004), retrospective cohort study (2b) [10]	Inclusion: IOP > 25 mmHg following congenital cataract surgery; Lensectomy < 20 y. Exclusion: Pre-existing glaucoma; History of trauma; Intraocular neoplasm, radiation therapy, anterior uveitis, anterior segment dysgenesis, ocular syndromes; PCG.	-	-	-	21 (21-0)	Cyclocryotherapy, TDLC, contact Nd:YAG laser cyclotherapy	IOP ≤ 21 mmHg with and without medications and no need for further surgery.	14	8.6 ± 7.6	- *Not specified*
Carter et al. (2007), Retrospective case series (4) [56]	Inclusion: Aphakic or pseudophakic glaucoma (unacceptable IOP combined with evidence of optic nerve damage) *Note: 3 eyes with unilateral cataract associated with PFV were included*; <16 y; Medical, and in some cases surgical therapy was performed in most patients prior to ECP treatment. Exclusion: PCG; Anterior segment dysgenesis; Follow-up < 1 y.	32.6	3.3	4.2	34 (32-2)	ECP (180°–270°)	IOP ≤ 24 mmHg and IOP decrease of more than 15% despite the addition of glaucoma medications, without sight-threatening complications.	53	3.7	Retreatment of eyes increased the overall success rate. *18%*
Schlote et al. (2008), retrospective cohort study (2b) [60]	Inclusion: Glaucoma in aphakia Note: Of the 21 patients with glaucoma in aphakia, lensectomy was performed for congenital cataracts (*n* = 11) and acquired cataracts (*n* = 10); IOP levels > 21 mmHg despite maximal medical therapy; Progression of glaucoma damage despite maximal medical therapy. Exclusion: Follow-up < 1 y.	31.1 ± 8.8	-	53.1 ± 23.6	21	TDLC	5 ≤IOP ≤ 21 mmHg with and without medication.	19 after 1 TDLC 48 after repeated TDLC	3.5 ± 2.4	Translimbal or pars-plana-modified GDD may be associated with a better long-term prognosis, and should be used prior to TDLC to avoid the increasing risk of hypotonia using a filtering procedure after cyclodestruction. *42.9%*
Cantor et al. (2018), Retrospective cohort study (2b) [55]	Inclusion: Glaucoma (not further defined) following cataract surgery; <16 y. Exclusion: -	34.1 ± 8.3	4.0 ± 2.5	6.0 ± 3.8	35 (27-8)	ECP (average 230° for first ECP, average of 151° for repeat ECP)	IOP ≤ 24 mmHg, no alternative glaucoma procedure following ECP, or occurrence of devastating complications With and without medications.	54 48 in a 75 in p Successful eyes had 1.1 ± 0.2 ECP treatments (average).	7.2 ± 3.6	The failure rate was not increased in pseudophakic patients relative to aphakic patients. *0%*
Glaser et al. (2019), retrospective cohort study (2b) [57]	Inclusion: Childhood glaucoma (not further defined) 80 eyes of 70 patients were included The most common glaucoma diagnoses were GFCS (60%), anterior segment dysgenesis (13%), and PCG (9%). The majority of eyes were aphakic (*n* = 45, 46%) or pseudophakic (*n* = 28, 35%). Exclusion: -	30.8 ± 7.9	-	9.5 ± 6.0 (for all eyes)	48 (60% of all eyes)	ECP	IOP ≤ 24 mmHg with and without medications, without any additional glaucoma surgery, without devastating complications, without progression to NLP visual acuity.	64 at 1 y 36 at 3 y 16 at 5 y (after single ECP) (for all eyes)	* 2.2	In multivariable analysis, of many risk factors considered, only a preoperative IOP < 32 mmHg was significantly associated with treatment success. *Not specified*

Abbreviations: GDx = glaucoma diagnosis; LOE = level of evidence; SD = Standard Deviation, GFCS: Glaucoma Following Cataract Surger; a = aphakic; p = pseudophakic; (*): median; mg = milligram; mL = millilitre; min = minutes; GDD = Glaucoma Drainage Device; IOP = Intra-Ocular Pressure; PCG = Primary Congenital Glaucoma; *n* = amount; PHPV = persistent hyperplastic primary vitreous; y = years; ECP = endoscopic cyclophotocoagulation;TDLC = transscleral diode laser cyclodestruction.

## Appendix A

Search conducted on 23 June 2019–last updated on 31 December 2021 in Pubmed (Medline): (“Glaucoma”[Mesh] OR Glaucoma[tiab]) AND ((“Aphakia, Postcataract”[Mesh] OR “Aphakia”[Mesh] OR Aphak*[tiab]) OR (“Pseudophakia”[Mesh] OR “Pseudophak”[tiab])) AND (“therapy”[Mesh] OR therap*[tiab] OR treatment[tiab] OR management[tiab]).

Search conducted on 23 June 2019–last updated on 31 December 2021 in Embase: Concept 1: (‘disease management’/exp OR ‘management’:ti,ab) AND Concept 2: (‘aphakic glaucoma’/exp OR ‘aphakic’:ti,ab) OR Concept 3: (‘pseudophakic glaucoma’ OR ‘pseudophak’:ti,ab) Combine.

Search conducted on 23 June 2019–last updated on 31 December 2021 in Web of Science Concept 1: TS = (Glaucoma AND Aphak* OR Pseuphak*) Concept 2: TS = (therap*OR treatment OR management) Combine.

Search conducted on 23 June 2019–last updated on 31 December 2021 in Cochrane: Concept 1: disease management AND Concept 2: aphakic glaucoma OR Concept 3: pseudophakic glaucoma.
